# Dynamic foraminal dimensions during neck motion 6.5 years after fusion and artificial disc replacement

**DOI:** 10.1371/journal.pone.0237350

**Published:** 2020-08-11

**Authors:** Sherwin Azad, Daniel Oravec, Timothy Baumer, Andrew Schildcrout, Parnell White, Azam Basheer, Michael J. Bey, Stephen W. Bartol, Victor Chang, Yener N. Yeni

**Affiliations:** 1 Bone and Joint Center, Department of Orthopedics, Henry Ford Health System, Detroit, Michigan, United States of America; 2 School of Medicine, Wayne State University, Detroit, Michigan, United States of America; 3 Department of Neurosurgery, Henry Ford Health System, Detroit, Michigan, United States of America; George Washington University, UNITED STATES

## Abstract

**Objective:**

To compare changes in foraminal motion at two time points post-surgery between artificial disc replacement (ADR) and anterior cervical discectomy and fusion (ACDF).

**Methods:**

Eight ACDF and 6 ADR patients (all single-level C5-6) were tested at 2 years (T1) and 6.5 years (T2) post-surgery. The minimum foraminal height (FH.Min) and width (FW.Min) achieved during neck axial rotation and extension, and the range of these dimensions during motion (FH.Rn and FW.Rn, respectively) were measured using a biplane dynamic x-ray system, CT imaging and model-based tracking while patients performed neck axial rotation and extension tasks. Two-way mixed ANOVA was employed for analysis.

**Results:**

In neck extension, significant interactions were found between year post-surgery and type of surgery for FW.Rn at C5-6 (p<0.006) and C6-7 (p<0.005), and for FH.Rn at C6-7 (p<0.01). Post-hoc analysis indicated decreases over time in FW.Rn for ACDF (p<0.01) and increases in FH.Rn for ADR (p<0.03) at the C6-7 adjacent level. At index level, FW.Rn was comparable between ACDF and ADR at T1, but was smaller for ACDF than for ADR at T2 (p<0.002). In axial rotation, differences were found between T1 and T2 but did not depend on type of surgery (p>0.7).

**Conclusions:**

Changes were observed in the range of foraminal geometry at adjacent levels from 2 years to 6.5 years post-surgery that were different between ACDF and ADR for neck extension. These changes are contrary to the notion that motion at adjacent levels continue to increase following ACDF as compared to ADR over the long term.

## Introduction

Anterior cervical spine surgery is commonly performed for the treatment of cervical spinal disease or deformity, accounting for more than 80% of the 1.3 million procedures performed on the cervical spine in the period between 2002 and 2009 [1]. Two procedures commonly performed are Anterior Cervical Discectomy and Fusion (ACDF) and Anterior Cervical Disc Replacement (ADR). It has been proposed that ACDF, and to a lesser extent ADR, may accelerate degeneration at adjacent levels leading to the development of adjacent segment disease (ASD) in the long term [2–5]. Randomized controlled trials (RCT) comparing the two procedures have generally found favorable outcomes with ADR [6–9]. A recent meta-analysis of 10 high-quality RCTs reported a relative risk of reoperation of 0.55 (95% CI, 0.35–0.85; P < 0.01) for patients treated with ADR compared to ACDF [10].

Adjacent segment disease may be affected by many intraoperative and patient specific factors [11], including the natural history of the adjacent level and natural progression of disease, the disruption of anatomy caused by the surgical procedure, and the biomechanical effect caused by the surgical device including the potential for malalignment [12]. It has been proposed that increased segmental motion at adjacent levels due to limiting motion at the operated segment (more so in a fused segment) is a major cause of accelerated degeneration at adjacent levels and development of ASD [2–5]. Despite favorable clinical outcomes for ADR over ACDF, the evidence connecting this result to the motion-limiting (for ACDF) or motion-preserving (for ADR) nature of the surgeries is lacking. In lack of such information further developmental efforts for design of new devices or treatment approaches may be misguided.

Previous efforts aiming to characterize the biomechanical basis of the adjacent level disease in cadaveric models have been challenged by difficulties in simulating the real world mechanical environment of a motion segment [13, 14], estimating post-operative behavior of the patient [15] and replicating biological processes around the implant. Standard radiographic [16, 17], magnetic resonance imaging (MRI) [18] and computed tomography (CT) [19–23] imaging techniques are limited to static neck positions, or ranges of motion that may not accurately represent the movements that the patient would normally make [24]. With the introduction of motion analysis systems utilizing dynamic biplane x-ray radiography together with computed tomography or magnetic resonance imaging, and computational techniques such as model based tracking, accurate three dimensional analysis of cervical spine motion during physiological neck motion tasks performed by live humans has become possible [25–27]. Using this technique, we have previously reported short-term (2 year post-surgery) differences in cervical spine intervertebral and neuroforaminal motion between ACDF and ADR patients during trials of neck axial rotation and flexion-extension [28, 29]. However, no physiologically accurate motion data comparing ACDF to ADR is available for a long term follow-up time point, greater than 5 years, i.e., at the onset of ASD.

Therefore, the objective of this study was to examine the cervical foraminal motion at two time points after ACDF and ADR surgeries using a biplane dynamic x-ray system and markerless tracking, which provides dynamic 3D images of the vertebrae during physiologic motion tasks. We hypothesized that changes in the adjacent segment foraminal motion over time would be different between ACDF and ADR surgeries, consistent with the notion that ASD is associated with a compensatory increase in the mobility of the adjacent segment after surgery. Our specific focus was on the motion of cervical neural foramina as measured by their dynamic dimensions during neck motion, with the understanding that they are a key factor in nerve root compression and thus appearance of clinical symptoms.

## Materials and methods

This study was approved by Institutional Review Board of the Henry Ford Health System (IRB #9165). Written consent was obtained from each subject. 23 patients undergoing single level anterior cervical surgery at the C5-6 level were enrolled in the study. The primary indication for surgery for all patients was cervical radiculopathy, and the type of surgery was chosen by each patient after discussion of treatment options, including the option for no treatment. Only patients with no complications or evidence of pseudarthrosis (ACDF group), device failure (ADR), or heterotopic ossification (ADR) were considered eligible for inclusion in the study. The exclusion of heterotopic ossification was to ensure that ADR patients would have mobile spines at the index level, as the focus of the study was on adjacent segment motion differences between the groups. In the end, none of the C5-6 single level ADR patients encountered during the study had appreciable evidence of heterotopic ossification. Initially there were 16 patients in the ACDF group (4 males, 12 females; 28–71 years) and 7 patients who underwent single-level cervical arthroplasty (ADR) (3 males, 4 females; 38–57 years). The prostheses used in the ADR group were the Prestige^®^ (Medtronic^®^, Minneapolis, MN) in two patients, and the Prodisc-C^®^ (Depuy-Synthes^®^, Raynham, MA) in all others. Motion testing occurred at two time points, T1 and T2, which corresponded to 2 years (mean 23.7 months, SD 7.6) and 6.5 years (mean 149.9 months, SD 25.1) post-operatively. At the long term follow up, data for 8 patients from the ACDF group, and for 6 patients from the ADR group were available; therefore, only these patients were included in the analysis. Their characteristics are summarized in Table 1.

**Table 1:** Patient characteristics.

	ACDF (n = 8)	ADR (n = 6)	p-value
**Age at operation (Years)**	42.5 ± 10.1	48.2 ± 7.5	p > 0.2
**Gender (Male/Female)**	4/4	2/4	p > 0.5
**Race (White/Black/Other)**	3/4/1	4/1/1	p > 0.2
**Time Post-op T1 (Months)**	26.0 ± 6.1	20.7 ± 8.8	p > 0.2
**Time Post-op T2 (Months)**	147.7 ± 34.3	148.4 ± 2.2	p > 0.9

Pre-operative MRI images were used to calculate the following radiographic indices at each levels of the C4-C7 segment to examine the pre-operative comparability of the two groups: Disc height, disc bulging, vertebral body width, vertebral canal width, Torg-Pavlov ratio [30] and the cervical stenosis grading scale proposed by Kang et al. (a grading scale of 0 to IV, higher grades being worse) [31]. None of these parameters were significantly different between the ACDF and ADR groups (p>0.08 to p>0.91).

Biplane x-ray images were acquired at 60 Hz during three trials of axial neck rotation and neck extension from a fully flexed, tucked chin posture, to full extension as previously described [28]. CT images were acquired including T1 to C3 levels for each patient using a LightSpeed16 system (GE Medical Systems, Milwaukee, WI), in axial mode with 0.625mm slice spacing, 0.25 x 0.25 pixel size, 130mm FOV and 512 x 512 acquisition matrix.

**Fig 1:**
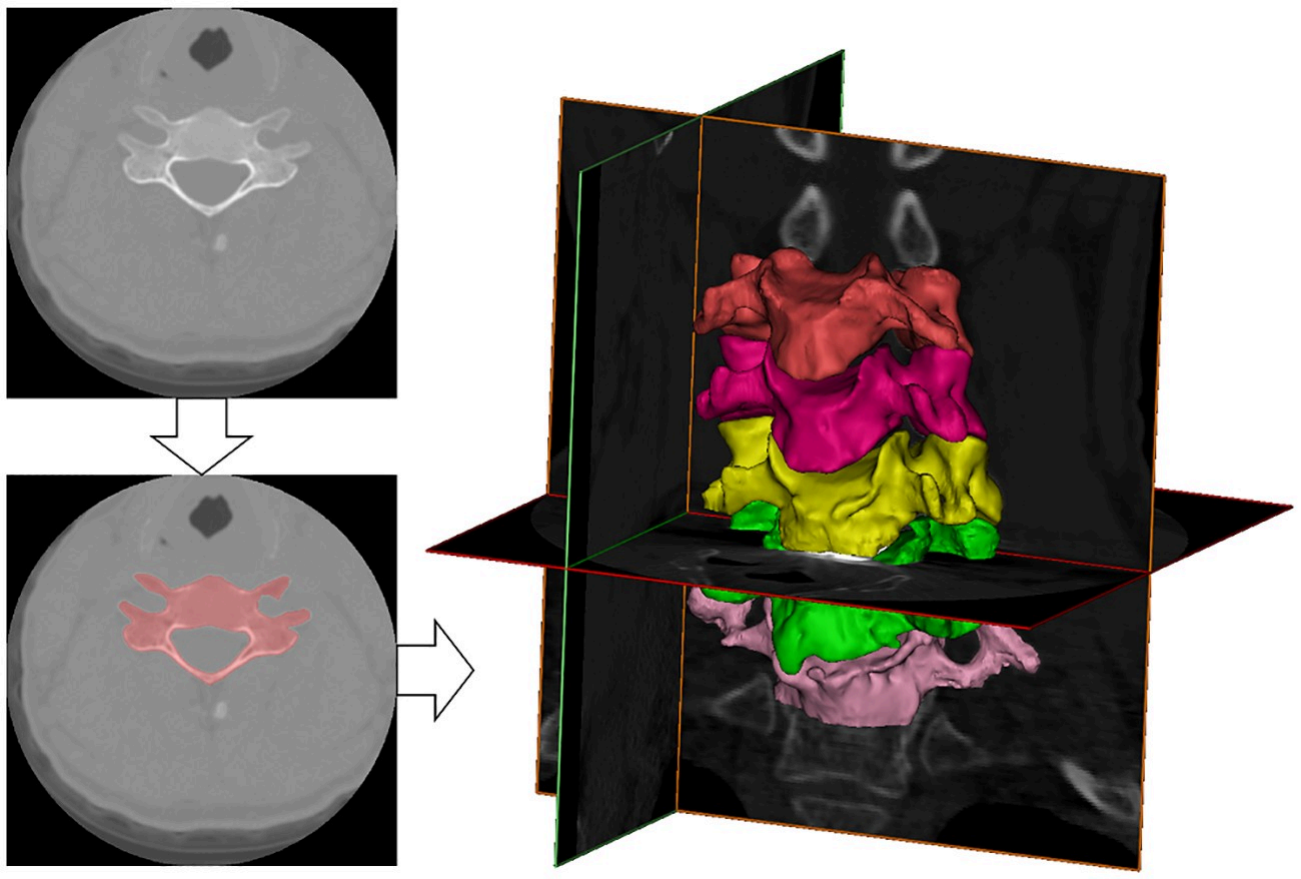
Construction of the motion model. A CT image of the cervical spine (top) is segmented, a bone mask is created (bottom; shaded area), and solid bone models were created for C4-C7 (right) using the volumetric mask, which was then used in model-based tracking.

**Fig 2:**
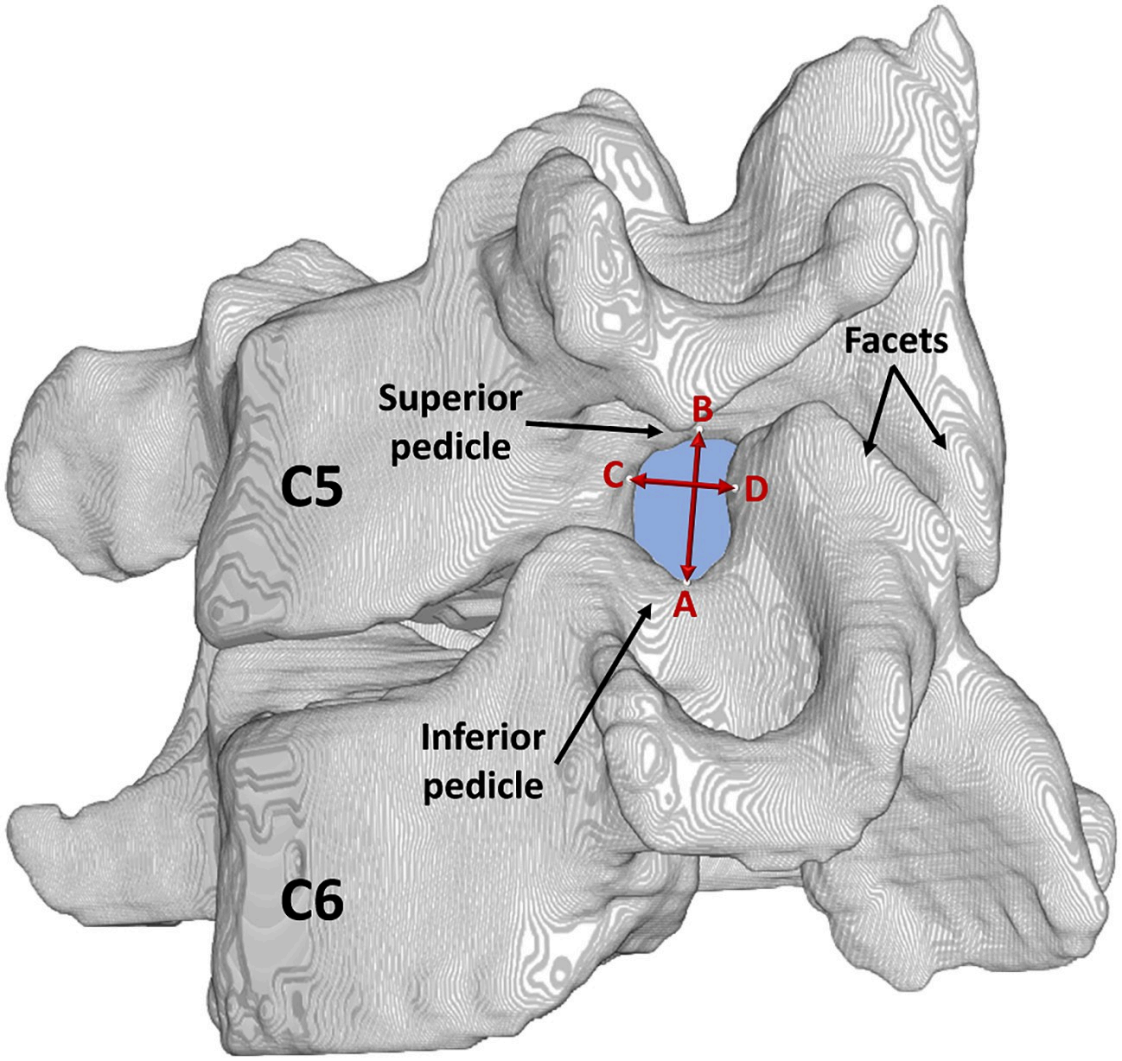
Landmarks. The most superior point of the inferior pedicle **(A)**, the most inferior point of the superior pedicle **(*B*)**, the anterolateral aspect of the superior vertebral body inferior notch **(*C*)**, and the posterolateral aspect of the inferior vertebral body superior notch **(*D*)** were marked to calculate foraminal height **(*A*↔*B*)** and width **(*C*↔*D*)** in 3D.

For foraminal dimension measurements, solid models were constructed using Mimics (Materialise, Plymouth, MI, USA), and four anatomical landmarks per foramen were identified on the 3D reconstructed model using custom software as detailed previously [32] (Fig 1). The landmarks describing the vertebral foramen consisted of the most superior point of the inferior pedicle, the most inferior point of the superior pedicle, the anterolateral aspect of the superior vertebral body inferior notch, and the posterolateral aspect of the inferior vertebral body superior notch. Foraminal height (FH) and width (FW) were then calculated as the 3D distance between the supero-inferior (SI) and antero-posterior (AP) markers, respectively [32] (Fig 2). Measurements were performed bilaterally at the C4-5 (upper adjacent), C5-6 (index) and C6-7 (lower adjacent) levels for each frame of data. Dynamic foraminal dimensions were quantified as the minimum (FH.Min, FW.Min) and the range (FH.Rn, FW.Rn) of foraminal height and width for each trial and then averaged over the three trials. The focus on range variables was for understanding the changes in the amount of foraminal motion, and the focus on the minimum variables was based on their representation of the potential for nerve root compression, with one assisting in the interpretation of the other.

In order to enhance the interpretation of foraminal motion results with reference to more commonly utilized range of motion (ROM) measures, the total ROM of the C4-C7 section was calculated for each participant in axial rotation and neck extension using methods identical to those described previously [28].

Analyses were performed separately for each motion segment. A two-way mixed ANOVA was employed, with one of foraminal motion (FH.Min, FH.Rn, FW.Min, FW.Rn) or C4-C7 ROM variables as the outcome, and post-surgical time-point (T1 and T2), surgery type (ACDF and ADR) and their interaction as the effect variables. The primary interest was in the interaction, as it would indicate a difference in the way foraminal motion changes over time between ACDF and ADR. When a significant interaction was found, the analysis proceeded separately for the two surgery types. JMP (v10, Cary, NC) software was used for the statistical analysis and significance was considered at p<0.05.

## Results

### Minimum foraminal dimensions during motion

No significant main effect, or interaction between time after surgery and surgery type was found for minimum foraminal dimensions (FH.Min, FW.Min), for both motion tasks and at all levels tested.

### Range of foraminal dimensions during motion

**Fig 3:**
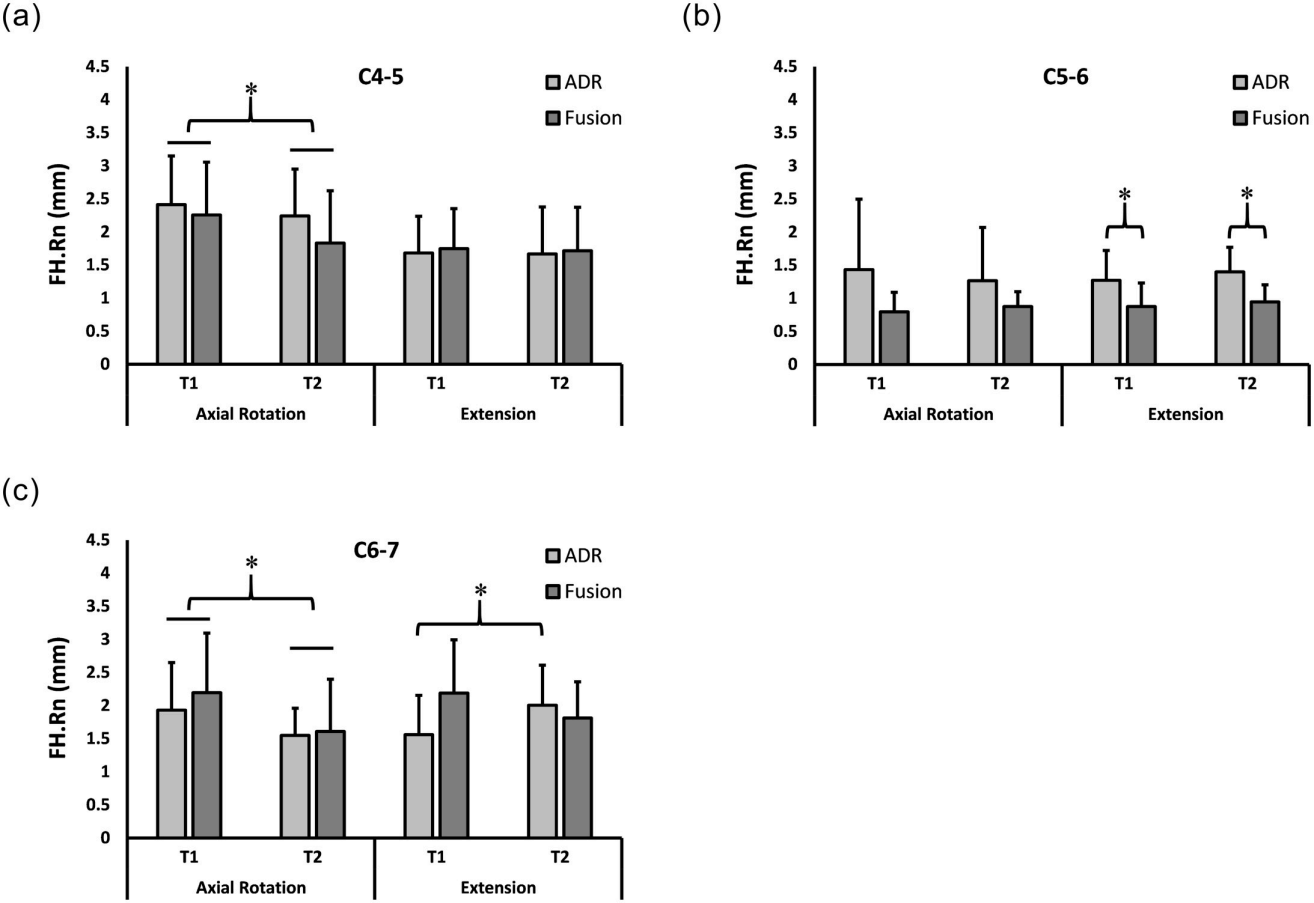
Foraminal Height Range (FH.Rn) at 2y and 6.5y. Range of foraminal height (FH.Rn) (Mean + SD) during neck axial rotation **(left)** and extension **(right)** movements at C4-5 **(A)**, C5-6 **(B)**, and C6-7 **(C)**. * Statistically significant difference.

**Fig 4:**
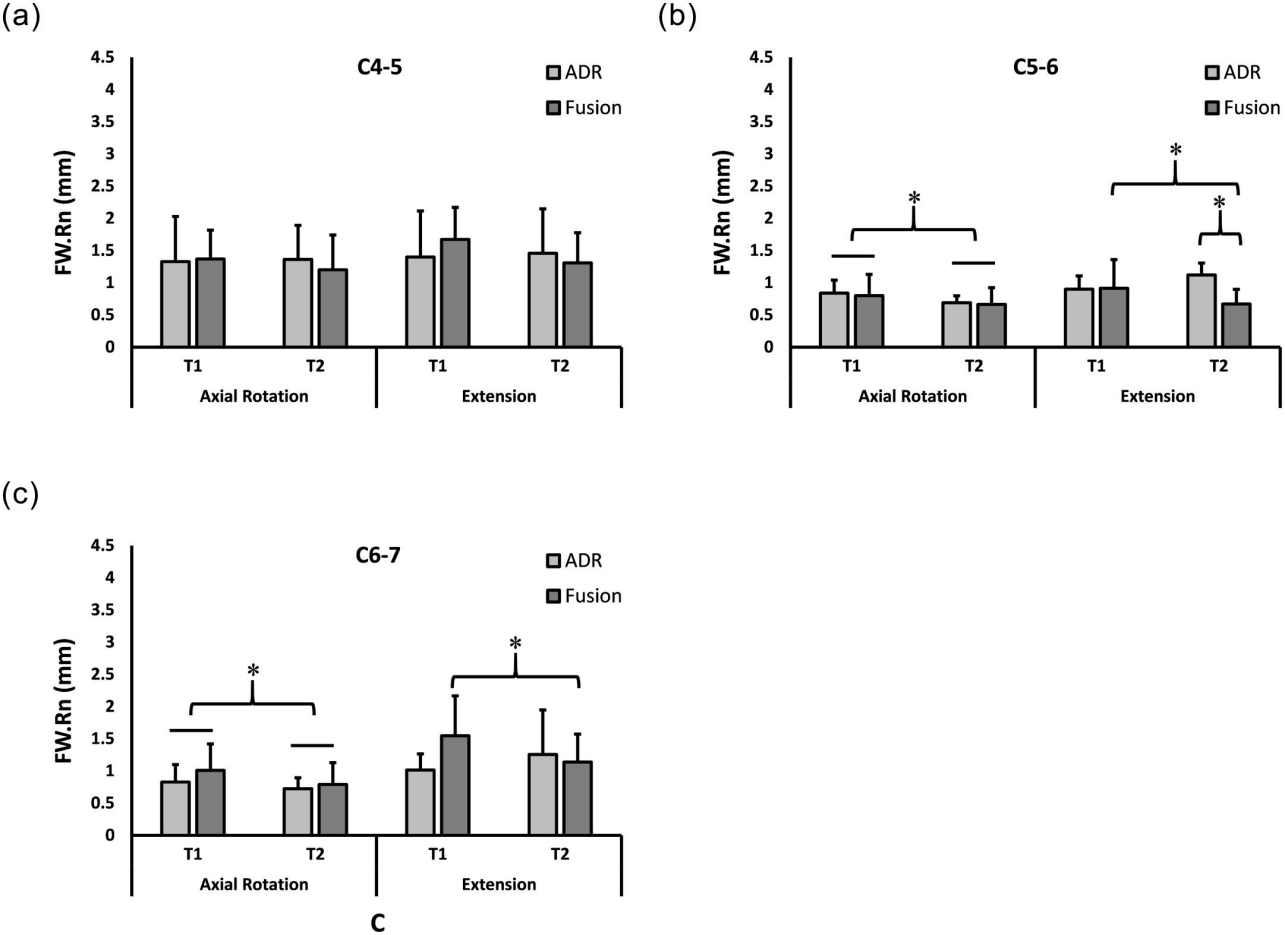
Foraminal Width Range (FW.Rn) at 2y and 6.5y. Range of foraminal width (FW.Rn) (Mean + SD) during neck axial rotation **(left)** and extension **(right)** movements at C4-5 **(A)**, C5-6 **(B)**, and C6-7 **(C)**. * Statistically significant difference.

The breakdown of dynamic range measurements by motion types, motion segments, surgery types and time points is presented as mean ± standard deviation for FH.Rn (Fig 3) and FW.Rn (Fig 4).

#### Upper adjacent level

No significant interaction was found for the C4-5 level for neck axial rotation or neck extension, suggesting that post-operative changes in foraminal dimensions over time were not different between fusion and ADR at the upper adjacent level.

Independently from surgery type, FH.Rn was smaller (p<0.03) at T2 than at T1 during neck axial rotation (Fig 3A).

#### Index level

No significant interaction was found between time after surgery and surgery type in neck axial rotation. Independent from surgery type, FW.Rn was smaller (p<0.009) at T2 than at T1 (Fig 4B).

Interaction was significant for FW.Rn during neck extension for the C5-6 index level (p<0.006). Post-hoc analysis indicated that FW.Rn was initially not different between ADR and ACDF (p>0.9), decreased by T2 for ACDF (p<0.01) while no demonstrable change was observed for ADR (p>0.1), and became less for ACDF than ADR at T2 (p<0.002) (Fig 4B). Independently from time of measurement, FH.Rn was greater for ADR than for fusion (p<0.03) in neck extension (Fig 3B).

#### Lower adjacent level

No significant interaction was found between time after surgery and surgery type in neck axial rotation. Independently from surgery type, FW.Rn and FH.Rn were smaller (p<0.05 and p<0.0001, respectively) at T2 than at T1 (Figs 4C and 3C).

Significant interactions were found for FW.Rn and FH.Rn in neck extension (p<0.005 and p<0.01, respectively). Post-hoc analysis indicated that FW.Rn was less at T2 than at T1 for ACDF (p<0.01) while no demonstrable change was observed for ADR (p>0.1) (Fig 4C). In contrast, FH.Rn was greater at T2 than at T1 for ADR (p<0.03) while no demonstrable change was observed for fusion (p>0.1) (Fig 3C).

#### C4-C7 total range of motion

No significant interaction was found between time after surgery and surgery type in the ROM for axial rotation or neck extension. Independently from surgery type, ROM was smaller at T2 than at T1 during neck axial rotation (p<0.001) and neck extension (p<0.03).

## Discussion

To our knowledge, this is the first in-vivo dynamic motion tracking study of ACDF and ADR patients at a long-term follow-up. With regards to our primary interest, we found changes in the range of foraminal motion over time that were different between the two surgery groups, during neck extension from a fully flexed chin tuck position. Particularly, the range of foraminal width decreased at the index and caudal adjacent levels in fusion; in contrast these changes were not observed in ADR. Additionally, the range of foraminal height increased at the caudal adjacent level in ADR.

Observations that the range of foraminal width decreased at the index level in fusion, and no such change was observed for ADR are consistent with motion-limiting and motion-preserving nature, respectively, of these surgeries. However, the decrease in adjacent segment foraminal motion observed for fusion, while an increase was observed for ADR at the long term follow up, was contradictory to our hypothesis and to the widely-held notion that development of adjacent segment disease is associated with increased motion at the adjacent level in compensation for immobilization of the index level in fusion. One possible explanation is that with spinal fusion, motion at adjacent segments is characterized by an initial period of hypermobility as found in shorter-term studies (1–2 years post-operative) [29, 33, 34], followed by degenerative changes that decrease mobility in the long-term [35]. Degenerative changes to any structure contributing to the geometry of neural foramina, such as the disc and the facet joint, may be involved in the development of hypomobility [36, 37]; however, changes to these structures were not examined in this study to substantiate this argument.

The differences between ACDF and ADR in the way foraminal motion changes over time were observed in neck extension only, while such differences were not demonstrable in axial rotation. This result suggests that neck extension is a sensitive task at eliciting changes in foraminal geometry that occur at levels adjacent to prior surgery, consistent with previous demonstrations that neck extension-flexion generally produces a large change in foraminal area [27, 32, 38–41]. However, it must be noted that ranges of foraminal dimensions observed in axial rotation were comparable to those in extension in this study, suggesting a large range of motion alone may not be indicative of the suitability of a task for assessment of the neck status. Neck extension-flexion activities have been reported to constitute a significantly larger portion of habitual neck motion activities than do axial rotation and lateral bending [42]. As such, the higher sensitivity of an extension task for surgery-caused changes in foraminal motion may be due to the relative accuracy of this task in reflecting the majority of habitual neck motions. These observations may have implications for the design of implants or treatment approaches to prioritize preservation of flexion-extension motion.

Although not different between surgery types, time-dependent changes were observed in other foraminal motion variables. A decrease in range of foraminal height at cranial adjacent level, a decrease in range of foraminal width at the treated level, and a decrease in range of foraminal width and height at the caudal adjacent level were observed, together with the decrease in total ROM, indicating an overall tendency for decreased cervical motion over time regardless of surgery. Sohn et. al showed in a cadaveric model that disc degeneration, as determined by height loss and disc bulge, was correlated with decreased foraminal width [43]. Splendiani et al. found that dynamic or “occult” stenosis of the neural foramen in symptomatic patients is associated with both a degenerated disc and a pathological facet joint in 100% of cases [44]. Humphreys et al. demonstrated that foraminal width decreases with aging, and this effect is particularly significant at the C6-C7 level [18]. As such, the changes in these variables observed between time points are likely due to aging and associated degenerative processes, and may be compounded by surgical intervention, but not attributable to a specific surgery type. Most notably, the changes in the range of foraminal dimensions over time, whether surgery type-dependent or not, were not accompanied by similar changes in minimum foraminal dimensions. This result suggests that the manifestation of changes in the range of dynamic foraminal dimensions after surgery is not necessarily an increased potential for nerve root compression via decreased minimum foraminal dimensions, although minimum foraminal dimensions can contribute to clinical symptoms independently from range of motion [45, 46].

There are several limitations with the current study, including the small number of patients studied, further reduced due to loss to follow-up. The patients lost to follow-up moved out of the system and apparently even out of state in the 4.5 years between the first and the second testing. They either could not be contacted due to loss of whereabouts or would not participate in the second testing due to the increased distance between their location and our institution. Patients were enrolled consecutively, and the two groups were not deliberately matched, and therefore group assignment depended on surgeon recommendation and patient choices consistent with the then-current practice, introducing selection bias. Additionally, pre-operative motion data were not collected. However, there were no significant demographic or radiographic differences observed between the two groups either at the time of surgery or at their two-year follow-up. Further, the design of the study was intended to compare data from the short (T1 = 2 y) and long term (T2 = 6.5 y) follow-ups so that the T1 point constitutes a baseline and each patient serves as their own control. As such, group differences were not of primary concern, rather their time-dependent changes were of concern. Within these limitations, we were able to demonstrate statistically significant effects. Given the longitudinal nature of the study, the long follow-up and the use of the state of the art motion imaging system, this study offers unique data regarding the course of adjacent segment motion following ACDF and ADR. Another limitation was a lack of direct assessment of degeneration and clinical outcomes to correlate with the motion data. However, this was not strictly necessary as our objective was to address a motion-related hypothesis. Though it could not be directly correlated to changes in motion parameters in the current study due to lack of T1 data, we did report the 6.5 year post-operative patient reported outcomes from this cohort in a recent study, where symptoms were none to moderate, and were not different between the ACDF and ADR groups [47]. As such, it is possible that foraminal motion changes precede development of severe symptoms. Nonetheless, future larger scale investigations should include radiographic signs of degeneration and clinical symptoms along with the motion information to better elucidate the differences in long-term sequelae of ACDF versus ADR. Surgery-dependent changes in foraminal motion with time were not accompanied by demonstrable changes in traditional total ROM parameters, indicating the importance of motion analysis finely focused on the area of mechanistic interest. A full comparison and correlation of segmental intravertebral (including rotations and translations) and foraminal motion was beyond the scope of this study, and will be the subject of future research aimed at understanding the relationship between potentially desirable foraminal motion characteristics and modifiable neck motion tasks.

## Conclusions

In contrast to our expectation, we found that over the long term, variation in the size of the foramen at adjacent levels decreased in fusion surgery, and in some cases had a mild increase following ADR, leading to a hypothesis that foraminal motion at levels adjacent to ACDF is characterized by an initial period of hypermobility, but eventually followed by hypomobility due to compensatory or degenerative mechanisms. We also found neck extension is a more sensitive test for surgery-dependent changes in foraminal motion at levels adjacent to prior fusion or arthroplasty. Future studies are warranted to more fully understand the time course of these changes and associated mechanisms to inform preventative and treatment options.

## Supporting information

S1 Data Data for Table 1. (XLSX)

S2 Data Foraminal dimension data for neck axial rotation. Used in Figs 3 and 4.
(XLSX)

S3 Data Foraminal dimension data for neck extension. Used in Figs 3 and 4.
(XLSX)

S4 Data Range of motion data for the C4-C7 section for axial rotation and neck extension. Used for statistics reported in the last paragraph of Results.
(XLSX)

S5 Data Pre-operative radiographic data. Used for statistics reported in the second paragraph of Methods.
(XLSX)
